# Protocol for a prospective, multicenter, parallel-group, open-label randomized controlled trial comparing standard care with Closed lOoP In chiLdren and yOuth with Type 1 diabetes and high-risk glycemic control: the CO-PILOT trial

**DOI:** 10.1007/s40200-024-01397-4

**Published:** 2024-03-07

**Authors:** Alisa Boucsein, Yongwen Zhou, Jillian J. Haszard, Craig A. Jefferies, Esko J. Wiltshire, Sara E. Styles, Hamish R. Crocket, Barbara C. Galland, Maheen Pasha, Goran Petrovski, Ryan G. Paul, Martin I. de Bock, Benjamin J. Wheeler

**Affiliations:** 1 Department of Women’s and Children’s Health, University of Otago, Dunedin, New Zealand; 2 Department of Endocrinology, Institute of Endocrine and Metabolic Diseases, Division of Life Sciences and Medicine, The First Affiliated Hospital of USTC, Clinical Research Hospital of Chinese Academy of Sciences (Hefei), University of Science and Technology of China (USTC), 230001 Hefei, Anhui China; 3 Biostatistics Centre, University of Otago, Dunedin, New Zealand; 4 Starship Child Health, Te Whatu Ora Te Toka Tumai Auckland, Auckland, New Zealand; 5 Liggins Institute, Department of Paediatrics, The University of Auckland, Auckland, New Zealand; 6 Department of Paediatrics and Child Health, University of Otago Wellington, Wellington, New Zealand; 7 Te Whatu Ora Capital, Coast and Hutt Valley, Wellington, New Zealand; 8 Department of Human Nutrition, University of Otago, Dunedin, New Zealand; 9 Te Huatakia Waiora School of Health, University of Waikato, Hamilton, New Zealand; 10 Sidra Medicine, Doha, Qatar; 11 Waikato Regional Diabetes Service, Te Whatu Ora Waikato, Hamilton, New Zealand; 12 Department of Paediatrics, University of Otago, Christchurch, New Zealand; 13 Te Whatu Ora Waitaha Canterbury, Christchurch, New Zealand; 14 Te Whatu Ora Southern, Dunedin, New Zealand

**Keywords:** Type 1 diabetes, Advanced hybrid closed loop, Automated insulin delivery, Artificial pancreas, Children and youth, Glycemic control

## Abstract

**Purpose:**

Advanced hybrid closed loop (AHCL) systems have the potential to improve glycemia and reduce burden for people with type 1 diabetes (T1D). Children and youth, who are at particular risk for out-of-target glycemia, may have the most to gain from AHCL. However, no randomized controlled trial (RCT) specifically targeting this age group with very high HbA_1c_ has previously been attempted. Therefore, the CO-PILOT trial (Closed lOoP In chiLdren and yOuth with Type 1 diabetes and high-risk glycemic control) aims to evaluate the efficacy and safety of AHCL in this group.

**Methods:**

A prospective, multicenter, parallel-group, open-label RCT, comparing MiniMed™ 780G AHCL to standard care (multiple daily injections or continuous subcutaneous insulin infusion). Eighty participants aged 7–25 years with T1D, a current HbA_1c_ ≥ 8.5% (69 mmol/mol), and naïve to automated insulin delivery will be randomly allocated to AHCL or control (standard care) for 13 weeks. The primary outcome is change in HbA_1c_ between baseline and 13 weeks. Secondary outcomes include standard continuous glucose monitor glycemic metrics, psychosocial factors, sleep, platform performance, safety, and user experience. This RCT will be followed by a continuation phase where the control arm crosses over to AHCL and all participants use AHCL for a further 39 weeks to assess longer term outcomes.

**Conclusion:**

This study will evaluate the efficacy and safety of AHCL in this population and has the potential to demonstrate that AHCL is the gold standard for children and youth with T1D experiencing out-of-target glucose control and considerable diabetes burden.

**Trial registration:**

This trial was prospectively registered with the Australian New Zealand Clinical Trials Registry on 14 November 2022 (ACTRN12622001454763) and the World Health Organization International Clinical Trials Registry Platform (Universal Trial Number U1111-1284-8452).

**Supplementary Information:**

The online version contains supplementary material available at 10.1007/s40200-024-01397-4.

## Introduction

Maintaining healthy glycemic control is challenging for people with type 1 diabetes (T1D). This is particularly so for children, and adolescents and young adults (henceforth referred to as “youth”) as their attempts to manage glucose levels to target can be limited by risk of hypoglycemia, fear of hypoglycemia, their own or their caregivers’ burden of care, and less treatment adherence e.g. in form of missed bolus insulin delivery and less frequent self-monitoring of blood glucose [1–3]. Accordingly, less than a third of children and youth with T1D achieve glycemic targets known to reduce the risk of developing diabetes complications and improve life expectancy [1, 4]. More efficacious and burden reducing therapies are clearly required to improve outcomes.

### Advanced hybrid closed loop

Advanced hybrid closed loop (AHCL), sometimes referred to as artificial pancreas or automated insulin delivery (AID), is currently the most advanced commercial insulin delivery technology for T1D, having evolved from early sensor augmented pump (SAP) therapy, and then hybrid closed loop [5, 6]. AHCL integrates real-time continuous glucose monitoring (rtCGM), continuous subcutaneous insulin infusion (CSII), and a computer algorithm to partially automate insulin delivery [7]. Several commercial AHCL systems are currently available and are defined by their ability to not only modulate basal insulin delivery but also provide varying degrees of automated boluses to correct high glucose levels [6]. Evidence from randomized controlled trials (RCTs) supports the potential of AHCL to safely improve glycemic outcomes, including improving HbA_1c_ while reducing hypoglycemia [8–14]. These data have now led to AHCL being the gold standard for insulin therapy for most people with T1D as recommended in current guidelines [15, 16]. However, these trials have often not been inclusive of those of non-European ethnicity nor varying socioeconomic status, and often excluded people with suboptimal glycemic control (particularly those with HbA_1c_ > 10% [86 mmol/mol]).

These individuals may in fact have the most to gain from automating insulin delivery and reducing diabetes burden. Current early data suggests this with a recent prospective 3-month single arm study among youth with out-of-target glycemia (average baseline HbA_1c_: 10.5% [91.2 mmol/mol]), highlighting very large HbA_1c_ improvements of on average 2.9% points (31.5 mmol/mol) following rapid onboarding of AHCL, with sustained improvements after 12 months of AHCL [17, 18]. Similar data are suggested for other AHCL systems, but have not been tested at extremes of elevated HbA_1c_ [10]. In addition, children and youth with out-of-target glucose control and from diverse backgrounds do appear interested in wearable diabetes technology and have engaged with past trials [19–22].

Equitably improving diabetes care for children and youth burdened by the requirements of diabetes care and the ability to achieve recommended glycemic targets is of paramount importance, given the full lifetimes of diabetes ahead of them, as well as the future substantial risk of long-term complications and mortality if their diabetes remains out of target [23, 24]. In New Zealand, only 2.3% of children and adolescents use AID, limited by absence of funding for CGM systems and restricted access to publicly funded insulin pump therapy, which disproportionally disadvantage youth and those of non-European ethnicity [25, 26]. The impact of out-of-target glycemia on sleep in youth is of further concern, given that suboptimal glycemic control has been demonstrated to impair various aspects of healthy sleep [27, 28], which in turn has been associated with impaired diabetes management and general health and wellbeing [29, 30]. The CO-PILOT trial is the first RCT comparing AHCL with standard diabetes care (multiple daily injections [MDI] and CSII) to test our hypothesis that AHCL will improve glucose control and reduce burden of care in children and youth with suboptimal glycemia naïve to AHCL and struggling to achieve recommended glycemic targets with traditional insulin therapies.

## Methods

### Study design

**Fig. 1:**
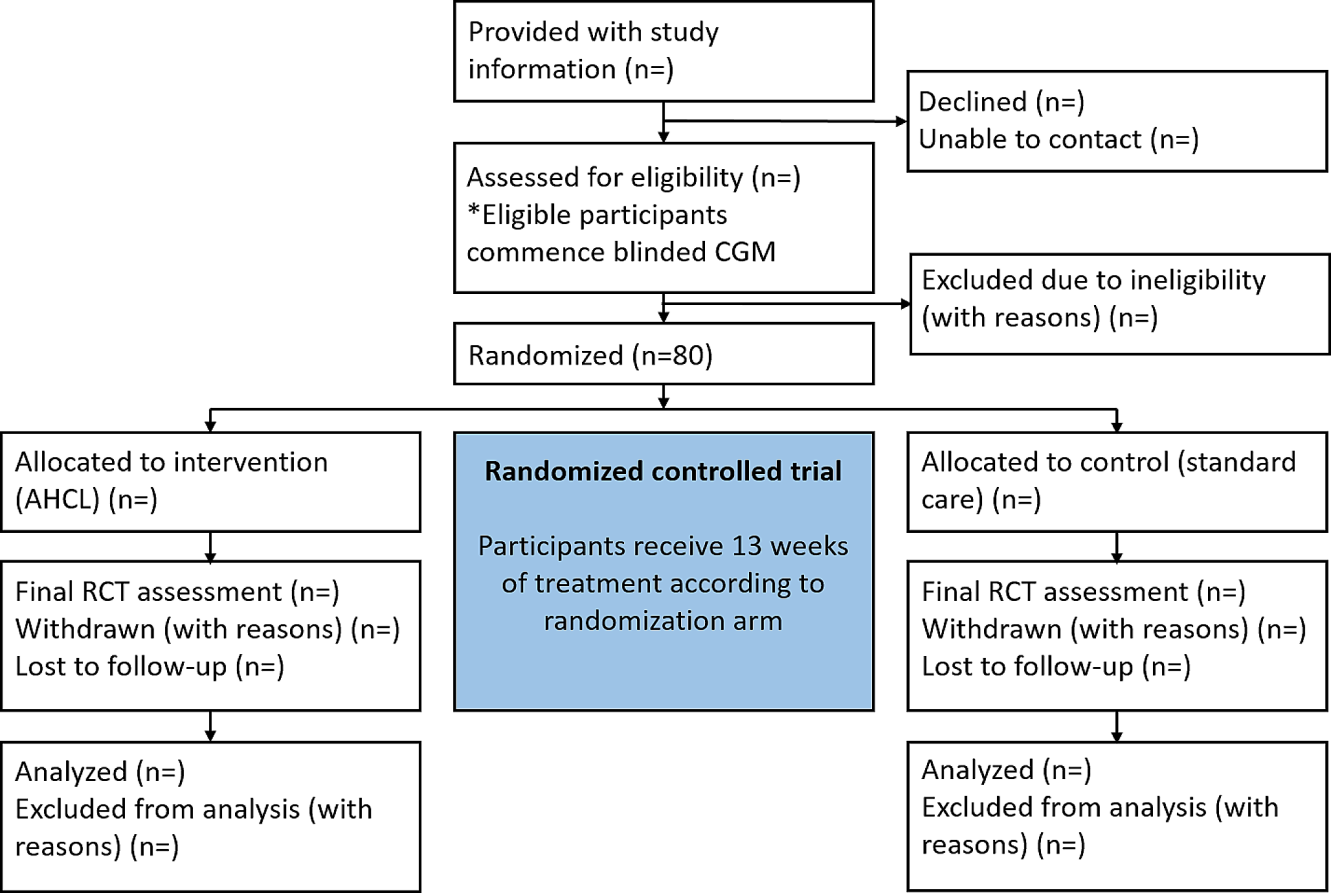
CO-PILOT study design. A total of 80 participants will be randomized; other sample sizes remain blank (n=) until trial completion

The CO-PILOT trial is comprised of a prospective, multicenter, parallel-group, open-label randomized controlled superiority trial evaluating the effectiveness and safety of AHCL compared to usual care over 13 weeks in 80 children and youth with T1D and suboptimal glycemic control (HbA_1c_ ≥ 8.5% [69 mmol/mol]), as shown in Fig. 1. A 2-week baseline data collection phase will precede the 13-week RCT, followed by a 39-week study continuation where control participants cross over to AHCL and all participants use AHCL until study conclusion. Total duration of participant involvement will be 54 weeks. The trial has been approved by the Southern Health and Disability Ethics Committee (Wellington, New Zealand; Ethics reference: 2022 FULL 13,508) and Māori (indigenous New Zealanders) Research Consultation Committees in each region.

### Sample size

Using a standard deviation of 23 mmol/mol and a within-person correlation of 0.6 [17, 31], a sample size of 64 (32 participants per group) would be required to determine a conservative difference in change in HbA_1c_ of 15 mmol/mol with 90% power to the alpha of 0.05 level (single arm data suggests > 15mmol/mol achievable in this population [17]). To account for 20% loss of data and participant attrition (inflated compared to other trials to allow for potentially greater loss of data due to the high-risk population), a sample size of 80 will be recruited (40 participants per group).

### Study population and recruitment

A total of 80 participants will be recruited at four diabetes centers across New Zealand (Dunedin, Christchurch, Wellington, and Auckland public hospitals) on a first-come first-serve basis. Participants will be patients receiving standard care through Te Whatu Ora– Health New Zealand diabetes services, identified by their usual endocrinology/diabetology team during routine clinical visits, invited to participate and provided with preliminary study information. Eligibility will be confirmed during screening visits. Inclusion criteria: aged 7 to 25 years inclusive; diagnosed with T1D as per American Diabetes Association classification [32] for ≥ 1 year; current HbA_1c_ ≥ 8.5% (69 mmol/mol); total daily dose (TDD) of insulin ≥ 8 units/day. Key exclusion criteria are: previous use of closed loop technology; pregnancy; use of medication indicative of diabetes complications (ACE inhibitors and statins permitted), systemic glucocorticoids, metformin, sodium-glucose cotransporter − 2 inhibitors, or glucagon-like peptide-1 receptor agonists; history of severe medical or psychiatric co-morbidities; any concomitant condition that would interfere with the study conduct or pose an unacceptable risk to participants. For diabetic retinopathy and other visual impairment, until future data on moderate to severe retinopathy is available, potential participants will be eligible if they meet the following criteria (based on internationally accepted grading criteria [33]): (A) Nil or minimal retinopathy– no restriction on study entry; (B) if Grade 1/mild retinopathy and HbA1c < 10% (86 mmol/mol)– no restriction to study entry; (C) if Grade 1/mild retinopathy and HbA_1c_ ≥ 10% (≥ 86 mmol/mol)– diabetic retinopathy screening to be performed during the study pre-screening phase. If subject remains at Grade 1/mild retinopathy and frequency of screening is deemed ≥ 1 year (indicating less clinical concern), inclusion criteria are met. If subject has progressed to moderate (Grade 2) or severe (Grade 3) diabetic retinopathy, to be excluded; (D) any diabetic retinopathy classed at or beyond moderate (Grade 2) or severe (Grade 3) non-proliferative retinopathy excluded; (E) history of severe visual impairment excluded. ISPAD guidelines [34] for diabetic retinopathy screening will be followed while in study care. These are: Screening from age 11 years with 2 to 5 years diabetes duration. Subsequent monitoring frequency 2- to 3-yearly (or as locally recommended/available).

### Study procedures

At screening/enrolment (Visit 1), prior to the start of any study-related procedures, written informed consent/assent will be obtained from participants and, if applicable, their legal guardians (for participants aged 7 to 15 years, and inclusive). Subsequently, demographic and anthropometric data, date of T1D diagnosis, current insulin therapy, prior CGM use, and co-morbidities will be recorded. Episodes of diabetic ketoacidosis (DKA) and severe hypoglycemia (defined as coma or convulsion requiring assistance from others) within the previous 12 months will be collected. Female participants of child-bearing potential will perform a urine pregnancy test. To confirm eligibility, HbA_1c_ will be measured by a calibrated point-of-care device. All participants will commence 2-week blinded CGM (Guardian™ Sensor 3 and Link 3 transmitter, Medtronic, San Francisco, CA) for continuous collection of baseline glycemic data. Participants will be instructed to perform self-monitoring blood glucose (SMBG) calibration at least 3 times daily during this phase (Contour Next Link 2.4 m, Medtronic). Questionnaires for participant-reported psychosocial and sleep outcomes will be administered, and habitual sleep patterns will be recorded using actigraphy prior to randomization.

### Meal announcement procedures

Participants will complete a dietitian-generated carbohydrate counting assessment tool to guide method of AHCL meal announcement (Suppl. Information S1) [35]. Based on assessment scores, participants will be instructed to announce AHCL meals by using either a flexible method of precise carbohydrate counting (flex method) or a preset of three fixed carbohydrate amounts for meals (fix method). These preset amounts for snacks, regular meals, and large meals will be personalized based on a 3-day food diary completed by participants.

Providing these two options for carbohydrate counting ensures knowledge of carbohydrate counting is not a potential barrier for using AHCL, as well as tests the philosophy that carbohydrate counting is required to obtain an insulin pump as part of this clinical trial. In support of this approach, recently published data from study investigators suggest that simplified fixed carbohydrate meal announcements are an effective alternative for AHCL users who struggle with precise carbohydrate counting [35].

### Randomization

Prior to study commencement, a randomization sequence for a 1:1 allocation with permuted blocks of random size, stratified by pre-randomization HbA_1c_ (8.5–11.2%, or ≥ 11.3% [69–99 mmol/mol, or ≥ 100 mmol/mol]) and age (aged 7–15 years, or aged 16–25 years) will be generated by a biostatistician using Stata 17.0 (StataCorp, College Station, TX) and imported into the Research Electronic Data Capture (REDCap) randomization module [36]. The REDCap project will subsequently be moved into production, rendering the randomization sequence inaccessible and preserving allocation concealment. Participants will be randomly allocated to the intervention arm (commence AHCL) or control arm (continue standard care) following baseline data collection using the randomization module. Participants and study staff will not be blinded to this intervention assignment.

### Study groups

#### Intervention arm

Participants allocated to intervention will undergo our previously published 72-hour rapid onboarding protocol (Wheeler-Petrovski initiation protocol) [17]. In brief, participants will be trained in the use of the MiniMed™ 780G AHCL insulin pump with Guardian™ 4 sensor and Guardian™ Link 4 transmitter (Medtronic, San Francisco, CA), and Accu-Chek™ Guide Link blood glucose meter (Roche, Basel, Switzerland) and commence sensor augmented pump therapy with predictive low glucose management (SAP + PLGM). Key system settings will be as follows: total daily basal dose reduced by 20% of participant’s previous dose if MDI, and identical if CSII; active insulin time (AIT) of 3.0 h; insulin-to-carbohydrate ratio (ICR) calculated using the formula 500/TDD and insulin sensitivity factor (ISF) in mmol/L calculated using the formula 100/TDD (1800/TDD for mg/dL), or participant’s established CSII ratios will be used. Suspend before low limit will be set at 58 mg/dL (3.2 mmol/L), with alert on low activated, and suspend before low alert and high glucose alert deactivated. Bolus increment will be set at 0.025 units. After using the system in SAP + PLGM mode for 72 h, the SmartGuard™ feature will be activated with the following settings: Algorithm target set at 100 mg/dL (5.5 mmol/L), or, if concerns of frequent hypoglycemia, 110 mg/dL (6.1 mmol/L) or 120 mg/dL (6.7 mmol/L) at investigator’s discretion; automated corrections on; AIT of 2.5 h. At investigator’s discretion, over time the above settings can be adjusted as required, and AIT will be adjusted to 2.0 h within the first weeks of the RCT as able. Participants will use the AHCL system continuously for 13 weeks.

The SmartGuard™ feature incorporates a model-based adaptive algorithm with proportional-integral-derivative insulin-feedback module, which automatically adjusts basal insulin delivery every 5 min to achieve glucose levels determined by a target sensor glucose (SG) value. The system can furthermore deliver an automated correction bolus up to every 5 min as required based on SG values and is designed to provide optimal results at a target of 100 mg/dL (5.5 mmol/L) and AIT of 2.0 h in most people [37]. For exercise or other high-activity occasions, a temporary SG target of 150 mg/dL (8.3 mmol/L) can be selected, during which automated bolus corrections are disabled.

**Fig. 2:**
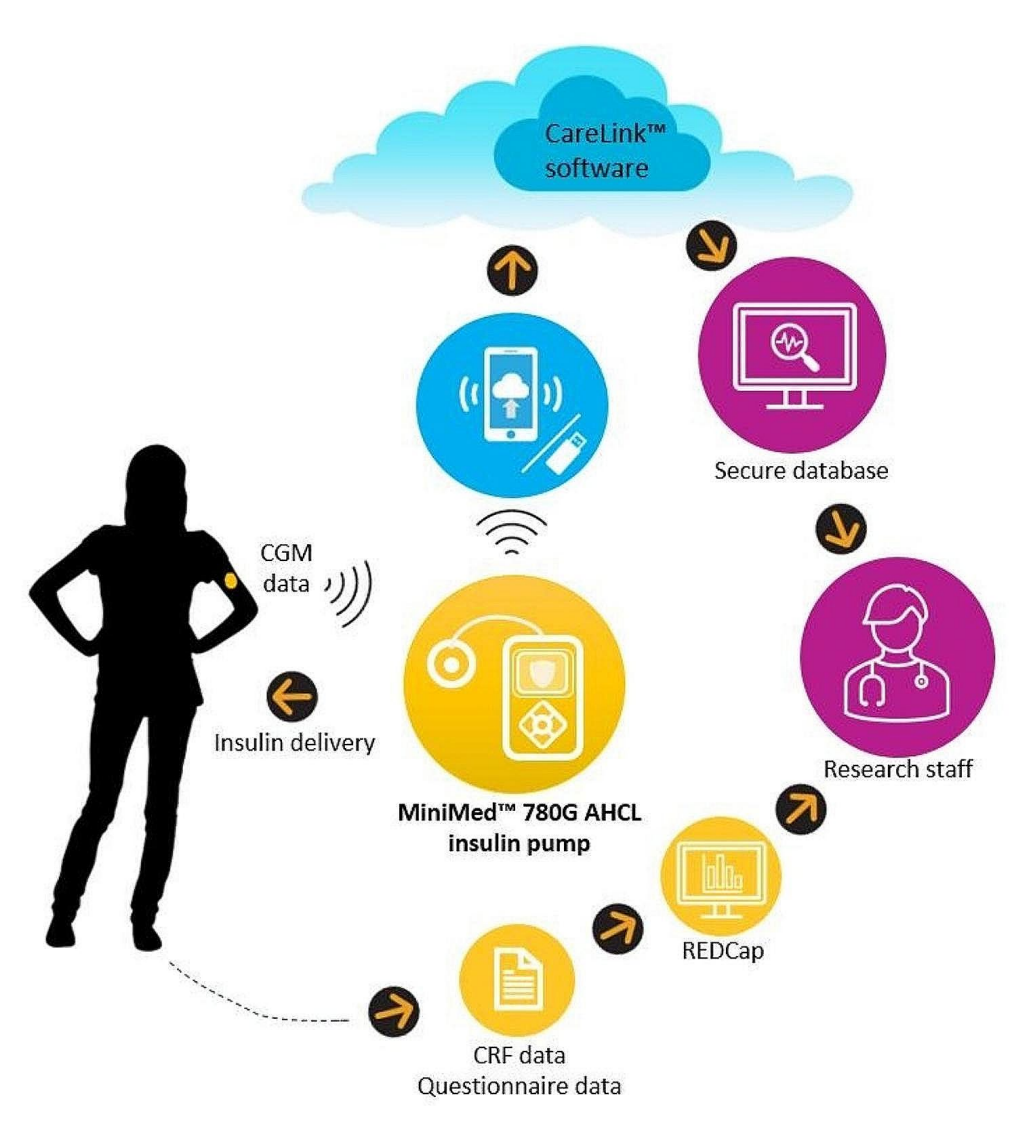
Data flow in the CO-PILOT study

Participants’ AHCL system data will be uploaded to CareLink™ Clinical Therapy Management Software (Medtronic, San Francisco, CA) using either the MiniMed™ Clinical App, or alternatively in case of app access issues a Bluetooth-enabled USB adapter (Blue Adapter, LG Innotek, Seoul, South Korea). Research staff will review data uploads remotely daily while participants are using SAP + PLGM therapy. Subsequently, remote data reviews and participant contact including setting adjustments, if required, will be daily for one week following SmartGuard™ initiation, then weekly for 4 weeks, then monthly until the end of the RCT. Setting adjustments will be verified by research staff during remote data reviews. Data flow in this study is depicted in Fig. 2.

#### Control arm

Control participants will continue their usual diabetes therapy (MDI or CSII [without automated insulin delivery]) throughout the 13-week RCT, and will receive remote contact from study staff at the same frequencies as described for the intervention group, including insulin dosing and glycemic data reviews, and management interventions as needed. Participants will repeat blinded CGM for continuous collection of end-of-study glycemic data during the final 2 weeks of the RCT. CGM use in the control group will be documented and reported, but no restrictions on CGM use are in place (other than those restricting AHCL/AID).

## Outcome measures

The primary outcome is the HbA_1c_ at the end of the 13-week RCT phase, comparing AHCL to usual care with adjustment for baseline HbA_1c_. Secondary outcomes will be assessed prior to randomization and during the final 2 weeks of the RCT. Recording of safety measures (DKA, severe hypoglycemia, hospitalizations, issues related to use of study devices) will be ongoing throughout the study. The schedule of primary and secondary outcome assessments is presented in Table 1.

**Table 1:** Study assessments

	Baseline	13 weeks	26 weeks	39 weeks	52 weeks
HbA_1c_	X	X	X	X	X
Glycemic metrics^a^	X	X	X	X	X
Psychosocial metrics^b^	X	X			
Sleep^c^	X	X			
Platform performance^d^	X	X	X	X	X
Safety^e^	X	X	X	X	X
Qualitative study^f^		X			

^a^Time in range 70–180 mg/dL (3.9–10 mmol/L); time in tight range 70–140 mg/dL (3.9–7.8 mmol/L); time < 70 mg/dL (3.9 mmol/L); time > 180 mg/dL (10 mmol/L); hypoglycemic events; day-time (0600–2359 h) and night-time (0000–0559 h) TIR. ^b^Hypoglycemia Fear Survey; Diabetes Treatment Satisfaction Questionnaires; Insulin Dosing Systems: Perceptions, Ideas, Reflections and Expectations. ^c^Pittsburgh Sleep Quality Index; Patient-Reported Outcomes Measurement Information System Sleep Disturbance and Sleep Impairment questionnaires; habitual sleep patterns via accelerometery. ^d^Alarm frequency; percentage time of SmartGuard™ use/sensor wear; SmartGuard™ exits. ^e^Diabetic ketoacidosis; severe hypoglycaemia; hospitalizations; any issues related to device use. ^f^Interviews will commence following completion of the 13-week RCT phase.

### HbA_1c_

HbA_1c_ will be measured by a calibrated point-of-care device (either DCA Vantage Analyzer, Siemens Healthcare Diagnostics, Swords, Ireland, or Cobas B 101, Roche Diagnostics, Rotkreuz, Switzerland). The same analyzer system will be used throughout the study at respective study centers. If HbA_1c_ at screening is above the detection range of the point-of-care device (> 14% [130 mmol/mol]), all subsequent HbA_1c_ tests for this participant will be performed by a formal diagnostic laboratory.

### Demographics

Demographic information including age, gender, ethnicity and address will be collected during screening. Participants can choose multiple ethnicities, however, each participant will be assigned to a single ethnicity following a prioritized system widely used in New Zealand with the hierarchical classification of Māori, Pacific, Other, and European [38]. Residential addresses will be used to assess socioeconomic status using the New Zealand Index of Deprivation 2018 which provides a deprivation score for small geographic areas defined by Statistics New Zealand. Deprivation data will be presented as quintiles (1–5) with 1 representing the least and 5 the most deprived areas in New Zealand [39].

### Anthropometry

Height and weight will be measured using calibrated instruments. Participants will be asked to wear light clothing and remove their shoes before measurements are taken. Height will be measured to the nearest 0.1 cm with a stadiometer and weight will be measured to the nearest 0.01 kg with a calibrated scale. Height and weight will be used to calculate body mass index (BMI) as per standardized formula. World Health Organization growth standards will be used for BMI z-scores [40].

### Glycemic metrics

CGM data will be collected from CareLink™ software and analyzed according to standardized CGM metrics for clinical care [41]. Time in range (TIR) will be recorded as the percentage of time with sensor glucose levels in the range 70–180 mg/dL (3.9–10.0 mmol/L); time in tight range (TITR) as the percentage of time with sensor glucose levels in the range 70–140 mg/dL (3.9–7.8 mmol/L); hypoglycemia as the percentage of time with sensor glucose levels below 70 mg/dL (3.9 mmol/L) and below 54 mg/dL (3.0 mmol/L); hyperglycemia as the percentage of time with sensor glucose levels above 180 mg/dL (10.0 mmol/L) and above 250 mg/dL (13.9 mmol/L). TIR will also be differentiated by day (0600–2359 h) and night (0000–0559 h).

### Psychosocial assessments

Psychosocial metrics will be collected through validated self-report questionnaires completed using paper forms and the order of administration will be standardized to increase reliability. Age-appropriate versions of questionnaires will be completed by children and adolescents, young adults, and, if applicable, guardians. All questionnaires will be administered in English. Where applicable, permissions and licenses for use of questionnaires were obtained.

The Hypoglycemia Fear Survey (HFS) is a measure of behaviors that people with T1D may engage in as a result of fear of hypoglycaemia and specific worries related to various aspects of hypoglycaemia [42]. Overall, higher scores reflect greater fear of hypoglycemia. A higher score on the Behavior Subscale reflects a greater tendency to avoid hypoglycemia and/or its negative consequences. A higher score on the Worry Subscale indicates more worry concerning episodes of hypoglycemia and its consequences. Participants aged 8 years and above will complete age-appropriate versions of the HFS. To accommodate the different number of items in the age-specific versions, the analysis will use mean item scores.

The Diabetes Treatment Satisfaction Questionnaire-status (DTSQs) and the DTQS-change (DTSQc) are self-report measures of a participant’s current treatment satisfaction [43, 44]. The DTSQc has been developed to overcome potential ceiling effects, where respondents score near-maximum satisfaction at baseline and would therefore show little or no improvement at follow-ups. The DTSQc will be administered only once, at the end of a study, in addition to the DTSQs at both baseline and end-of-study. Participants aged 13 years and above will complete age-appropriate versions of the DTSQs and DTSQc.

The Insulin Dosing Systems: Perceptions, Ideas, Reflections and Expectations (INSPIRE) questionnaire measures expectations of automated insulin delivery systems in people with T1D [45]. Different questionnaires exist for baseline and post-intervention assessment, which assess impacts on perception of glycemic control, activities, health complications, individual and family quality of life, and usability of the device. The INSPIRE questionnaire will be completed prior to randomization by all participants aged 8 years and above, however, only those randomized to the intervention arm will complete the post-intervention assessment at the end of the RCT.

### Sleep assessments

Sleep will be assessed using subjective and objective methods. Participants aged 13 years and above will complete the Pittsburgh Sleep Quality Index (PSQI) to assess sleep quality and timing during the previous one month to discriminate between good and poor sleep [46]. Originally developed for adults, two items related to sleeping with a bed partner will be excluded for adolescents, as previously described [31]. The PSQI generates 7 domains for subjective sleep quality, sleep latency, sleep duration, sleep efficiency, sleep disturbance, sleep medication, and daytime dysfunction, and a global score. In adolescents, scoring for the sleep duration domain will be adjusted according to participant age to reflect the number of hours of sleep recommended as different to adults [47].

Participants will also complete the Patient-Reported Outcomes Measurement Information System (PROMIS) Sleep Disturbance (SD) and Sleep-Related Impairment (SRI) short form questionnaires to assess qualitative aspects of wake function and sleep [48]. These are generic measures for gauging the severity of sleep-wake problems on a continuum, applicable across a range of health conditions. Participants aged 8 years and above will complete age-appropriate versions of the PROMIS questionnaires.

To objectively evaluate habitual sleep and wake patterns across repeated day-night cycles, participants of all ages will wear a 3-axis accelerometer (AX3, Axivity, Newcastle, UK) on the non-dominant wrist continuously for up to 7 days and 8 nights during baseline data collection and in the final 2 weeks prior to primary outcome collection. In participants aged 7–12 years, the device will additionally be worn by a guardian to assess their sleep. This device detects movement, vibrations and orientation changes at high precision and incorporates temperature and ambient light sensors to reinforce detection of periods of wear. Outputs will be processed using the count-scaled algorithm written in MatLab (Mathworks, Natick, MA, USA) to produce sleep variables related to sleep timing, quantity, quality and variability [49]. Processing will occur in either automatic mode using “time flags” for sleep onset (bedtime) and sleep offset (wake time), or for difficult files, processed under a “manual” mode where sleep onset and offset are visually identified from the activity outputs.

### Platform performance

AHCL system characteristics will be extracted from CareLink™ software and system settings, insulin delivery distribution (e.g., TDD, percentage insulin delivered through automation), system performance (e.g., alarm frequency, percentage time spent in SmartGuard™, sensor wear percentage time), and markers of therapy adherence (e.g., frequency of infusion set changes, bolus frequency) at the end of the RCT will be reported. Episodes of SmartGuard™ exits will be assessed.

### Qualitative study

It is important to understand participants’ lived experiences with AHCL technology. Semi-structured one-to-one interviews (in-person or via videoconference) will be conducted with 10 to 15 participants aged 13–25 years, and 10 to 15 guardians of participants aged 7–15 years using purposive sampling. Interviews will occur during the extension phase following the completion of the 13-week RCT, and interviewees will be interviewed after a minimum of one month of AHCL use. Interviews will last approximately 60 min and will be digitally recorded and transcribed verbatim for analysis in NVivo (Lumivero, Denver, CO). Thematic analysis will be performed to identify barriers and facilitators of AHCL use.

### Safety

Participants will be instructed to inform study staff immediately of the occurrence of any adverse events related to study devices (e.g., cutaneous events), or any serious adverse events (SAE) both related and un-related to study devices (e.g., hospitalization, DKA, severe hypoglycemia). Clinical investigators will advise about medical treatment, if necessary. All SAE will be reported to the lead investigator immediately after being reported to research staff or within one day of occurrence.

Technical support will be provided to all participants as needed while using AHCL from study staff and through the device manufacturer’s technical helpline. Any device deficiencies (medical device inadequacies with respect to its identity, quality, durability, reliability, safety or performance, notably including use errors) will be recorded and reported to the device manufacturer. A device deficiency that could have led to a SAE if circumstances had been less fortunate will be managed as a device-related SAE.

### Statistical analysis

Statistical analyses will be performed using the up-to-date version of specialist statistical software (R, SAS, or Stata), and results will be reported in line with the CONSORT statement. The biostatistician will be blinded to intervention assignment and will use non-informative group codes until all analyses are completed. Appropriate descriptive statistics will be presented for all variables, including means and standard deviations for continuous normally distributed variables and counts and percentages for categorical variables. The primary analysis will follow the intention-to-treat principle, with participants analyzed as per randomization allocation. A secondary per-protocol analysis will also be conducted where only those participants in the intervention group using SmartGuard™ for at least 70% of the time will be included. An alpha of 0.05 will be considered statistically significant. Mixed effects regression models will be used to estimate mean differences in outcomes, with 95% confidence intervals and p-values between groups. Models will be adjusted for baseline and stratification variables. A random effect for site will be included. There will be no adjustment for multiple comparisons. Residuals of all models will be plotted to assess whether homoscedasticity assumptions are met. If necessary, outcome variables may be log-transformed or quantile regressions undertaken.

## Discussion

Despite ongoing iterative advances in diabetes management, children, adolescents and young adults with T1D remain at high risk for out-of-target glycemia [4, 50–52]. This results in greater experience of both acute and chronic diabetes complications, as well as an increased likelihood of experiencing considerable diabetes- and non-diabetes-related burdens, including diabetes distress, stigma, psychiatric morbidity, and family/inter-personal conflict [53, 54]. AHCL, already the gold standard therapy for people with healthier glycemia [55–57], remains under-utilized in those with out-of-target control, which may in fact be used as a reason for exclusion from technology use, both clinically and in all randomized trials to date [9–14]. Our hypothesis is that for children and youth not meeting internationally recommended glycemic targets, AHCL should be the gold standard therapy and is likely to provide benefits for most with regards to burden reduction, psychosocial wellbeing, and glycemia. To our knowledge, the CO-PILOT trial is the first RCT to elucidate the impact of AHCL among this largely technology naïve, high-risk child and youth population (particularly those with HbA_1c_ > 10% [86 mmol/mol]) compared with traditional therapies.

Important strengths of the CO-PILOT trial are the outpatient structured training approach with intentional recruitment of a child and youth population with: (1) elevated HbA_1c_ ≥ 8.5% (≥ 69 mmol/mol); (2) diverse ethnic and socioeconomic backgrounds; (3) current MDI and CSII use without AID exposure; (4) opportunity for rapid outpatient on-boarding to automation with simplified carbohydrate counting options to avoid traditional potential delays around training location and carbohydrate counting education. Other strengths are the study duration with the continuation phase providing access to all participants out to week 54 as well as the in-depth psychosocial assessments (including sleep quality and qualitative work) to provide a greater understanding of AHCL acceptability and benefit. Limitations of the study include complexities of the utilized blinded CGM system which tends to lose data above 400 mg/dL (22.2 mmol/L) and which if anything leads to under-estimation of time above range at baseline and in the control group, but does not impact the HbA_1c_ primary outcome. Real-world clinic support may also differ from what is provided during the study, however, all efforts have been made to ensure translatable management including comparable contact with control participants, outpatient-based training and management, as well use of telehealth/phone-based remote contact.

In conclusion, in the CO-PILOT trial we aim to show that AHCL will have both immediate and long-term health benefits for participants and, where applicable, their caregivers. These results have the potential to translate into global impact by providing gold standard RCT evidence establishing the effectiveness and safety of AHCL in children and youth struggling with diabetes management and burden. This evidence is essential if we are to establish AHCL as the gold standard for glucose lowering therapy in people with T1D regardless of pre-conceptions related to their prior glucose control.

### Electronic supplementary material

Below is the link to the electronic supplementary material.

Supplementary Material 1
