# Supplementary material for: Protocol for a prospective, multicenter, parallel-group, open-label randomized controlled trial comparing standard care with Closed lOoP In chiLdren and yOuth with Type 1 diabetes and high-risk glycemic control: the CO-PILOT trial
Source: J Diabetes Metab Disord. 2024 Mar 7;23(1):1397–407. doi: 10.1007/s40200-024-01397-4 (PMC11196497; doi:10.1007/s40200-024-01397-4)
Supplement: Supplementary file 1 — Supplementary Material 1 [file 40200_2024_1397_MOESM1_ESM.docx]

**Protocol for a prospective, multicenter, parallel-group, open-label randomized controlled trial comparing standard care with Closed lOoP In chiLdren and yOuth with Type 1 diabetes and high-risk glycemic control: the CO-PILOT trial**

**Journal of Diabetes & Metabolic Disorders**

# **Authors**

Alisa Boucsein, PhD, Yongwen Zhou, MD, Jillian J. Haszard, PhD, Craig Jefferies, MD, Esko Wiltshire, MD, Sara E. Styles, PhD, Hamish Crocket, PhD, Barbara Galland, PhD, Maheen Pasha, MSc, Goran Petrovski, PhD, Ryan G. Paul, PhD, Martin I. de Bock, PhD, Benjamin J. Wheeler, PhD

**Corresponding author**

Professor Benjamin J. Wheeler, [ben.wheeler@otago.ac.nz](mailto:ben.wheeler@otago.ac.nz)

Department of Women’s and Children’s Health, University of Otago, Dunedin, New Zealand

**Supplementary Information S1:** Carbohydrate counting assessment tool for meal announcement with MiniMed™ 780G.

| **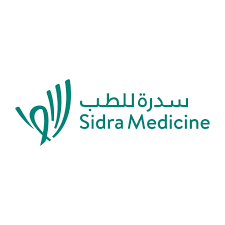 Carbohydrate Counting Assessment Tool for**  **Meal Announcement with MiniMed 780G** |
| --- |

**Participant Initials: …………………….…………… Study ID: …………..………………………………..**

**Date: ………………………………………..**

**Please complete the following questions:**

| 1. **Fast Acting Insulin** |
| --- |

**1. Using your own ratios: ICR……..…g; ISF…..……mmol/L; Target …..……mmol/L**

Please calculate the following:

The bolus insulin dose before the breakfast meal if:

1. The total CHO in the meal is 50g, the blood glucose reading is 6.0mmol/L:

………………………………………………………………………………………………………………………

1. You are not going to eat, the blood glucose reading is 15mmol/L:

………………………………………………………………………………………………………………………………….

1. The total CHO in the meal is 5 g, blood glucose reading is 6.0mmol/L:

………………………………………………………………………………………………………………………

Score: ____/1.5

**2. The main purpose of my fast-acting Insulin (Novorapid, Humalog or Apidra) is to stop my blood sugars from rising too high when I eat… (Choose one answer)**

⬜ Fat ⬜ Protein ⬜ Carbohydrate ⬜ All the above

Score: ____/1.0

**3. When you eat foods with carbohydrates, what is the best time to inject your fast-acting insulin? (Choose one answer)**

⬜ 1 hour before eating ⬜ Just before you start eating ⬜ After you finished eating

Score: ____/1.0

| 1. **Carbohydrate Counting** |
| --- |

**4. Which of these foods contain carbohydrates, meaning that you would need to count them when taking insulin? (Choose all that apply)**

⬜ Cornflakes ⬜ Nuts ⬜ Rice ⬜ Cheese ⬜ Tomato

⬜ Pasta ⬜ Bananas ⬜ Chicken ⬜ Eggs ⬜ Bread

Score: ____/2.5

**5. Imagine your blood glucose is normal and you want to eat 2 slices of toast (60g), 1 boiled egg and 1 cup milk (240ml) at breakfast. Calculate how many carbohydrates are in this meal.**

……………………………………………………………………………………………………………… Score: ____/1.0

**6. How many carbohydrates do you think are in each one of these snacks?**


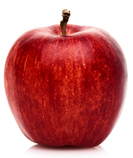

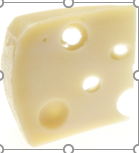


Apple (130g) is ..………g Cheese (25g) is ….……g Score: ____/1.0

**7. How many carbohydrates are in one cup of cooked white rice (160g)?**

……………………………………………………………………………………………………………………… Score: ____/1.0


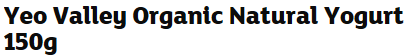
**8. How many carbohydrates are in this yogurt (weight 150g)?**


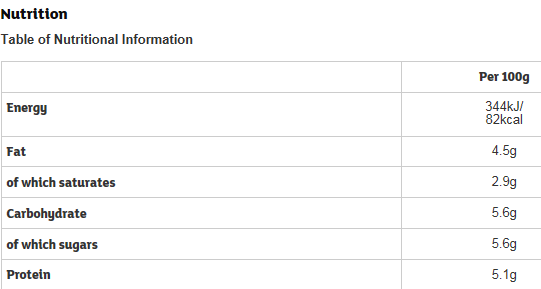


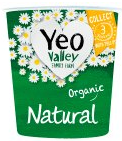


⬜ 51g ⬜ 8.4g ⬜ 15g Score: ____/1.0

| 1. **Carbohydrate Count Readiness Assessment**   **To be completed by the Registered Dietitian** |
| --- |

**Total score: ____/10.0**

⬜ **Score >=7:** Meal Announcement with precise carbs (demonstrates carbohydrate counting)

⬜ **Score 3.5 – 6.5:** Needs further teaching (to evaluate which group belongs)

- **Refer to Dietitian for Zoom intervention**

⬜ **Score <=3:** Fixed carbs, Meal Announcement with three pre-sets of carbohydrates

**Researcher’s Name: ………………….……………………....…… Date: ………………………………**
